# Model-based patient matching for in-parallel pressure-controlled ventilation

**DOI:** 10.1186/s12938-022-00983-y

**Published:** 2022-02-09

**Authors:** Jin Wai Wong, Yeong Shiong Chiew, Thomas Desaive, J. Geoffrey Chase

**Affiliations:** 1 School of Engineering, Monash University Malaysia, Selangor, Malaysia; 2 GIGA-In Silico Medicine, University of Liege, Liege, Belgium; 3 Centre for Bioengineering, University of Canterbury, Christchurch, New Zealand

**Keywords:** Parallel ventilation, Co-ventilation, Decision support, Model-based method

## Abstract

**Background:**

Surges of COVID-19 infections have led to insufficient supply of mechanical ventilators (MV), resulting in rationing of MV care. In-parallel, co-mechanical ventilation (Co-MV) of multiple patients is a potential solution. However, due to lack of testing, there is currently no means to match ventilation requirements or patients, with no guidelines to date. In this research, we have developed a model-based method for patient matching for pressure control mode MV.

**Methods:**

The model-based method uses a single-compartment lung model (SCM) to simulate the resultant tidal volume of patient pairs at a set ventilation setting. If both patients meet specified safe ventilation criteria under similar ventilation settings, the actual mechanical ventilator settings for Co-MV are determined via simulation using a double-compartment lung model (DCM). This method allows clinicians to analyse Co-MV in silico, before clinical implementation.

**Results:**

The proposed method demonstrates successful patient matching and MV setting in a model-based simulation as well as good discrimination to avoid mismatched patient pairs. The pairing process is based on model-based, patient-specific respiratory mechanics identified from measured data to provide useful information for guiding care. Specifically, the matching is performed via estimation of MV delivered tidal volume (mL/kg) based on patient-specific respiratory mechanics. This information can provide insights for the clinicians to evaluate the subsequent effects of Co-MV. In addition, it was also found that Co-MV patients with highly restrictive respiratory mechanics and obese patients must be performed with extra care.

**Conclusion:**

This approach allows clinicians to analyse patient matching in a virtual environment without patient risk. The approach is tested in simulation, but the results justify the necessary clinical validation in human trials.

**Supplementary Information:**

The online version contains supplementary material available at 10.1186/s12938-022-00983-y.

## Background

Surges of COVID-19 infections have prompted extreme demand for mechanical ventilation (MV). Up to 30% of COVID-19 hospitalised patients are likely to require ventilator support [1], creating severe demand spikes. Thus, the volume of patients susceptible to COVID-19 is much greater than the number of ventilators available in most hospitals. As a result, numerous hospitals have been confronted with the inadequacy in providing sufficient ventilators to support respiratory failure patients [2–4]. Due to the high cost and temporal demand, it is not feasible for a hospital to acquire and maintain this surge capacity of ventilators. Equally, long lengths of MV treatment means hospitals may see shortfalls in ventilators, leading to rationing of care and significant clinical stress, including triaging patients to receive MV care preferentially to others [4].

One solution in shortage of ventilators is to perform in-parallel co-mechanical ventilation (Co-MV) to two or more patients simultaneously. Co-MV, where patients breathe together, has been tested experimentally [5–7] and clinically [8]. To personalise MV settings for each patient, flow restrictors can individualise the flow and volume of air delivered to each patient. The use of high-efficiency particulate air (HEPA) filters can help limit infectious cross-contamination [9]. In addition, installing flow sensors along with additional displays will enable clinicians to monitor and personalise each patient’s care [10]. However, Co-MV can offer significant risks due to the inability to individualise MV settings and monitor the personal condition of each patient [11], increasing risk to patients from both over, and under, ventilation [12].

Despite various designs for Co-MV circuits, safety issues still arise whenever clinicians are unsure about the pairing criteria of the patients, and determining the corresponding MV settings for successfully implementing Co-MV. This issue is mainly due to the inability to compensate for variability in patient-specific size and respiratory system mechanics, which vary over the course of disease [13, 14]. To date, no published studies provide a guideline regarding patient matching and MV settings for Co-MV. Trial-and-error methods are impractical as the repetitive process of switching patients and circuits will increase the risk of other infections and ventilator-induced lung injury (VILI) when patients are mismatched. Thus, it is important to ensure safety if Co-MV is applied.

One proposed Co-MV method is the in-parallel Co-MV. For this Co-MV method, patients must be completely sedated during Co-MV to prevent any spontaneous breathing which would impact on both care and model-based identification of patient-specific respiratory mechanics [15–19]. Similarly, a pressure control (PC) ventilation mode should be used, as it strictly controls peak inspiratory pressure (PIP) and the positive end-expiratory pressure (PEEP) to minimise barotrauma risk, but cannot control peak inspiratory volume [20]. Other key Co-MV parameters include setting respiratory rate (RR) and the ratio of inspiration time to expiration time (I:E).

The major concern of Co-MV in PC mode is the shared tidal volume (VT) delivered to each patient. The distribution of VT depends solely on the patient-specific respiratory mechanics, which vary over time and with patient care [21, 22]. Patients with different respiratory mechanics will receive different VT in proportion to these values, and in particular, in proportion to their relative respiratory elastance. A patient with higher respiratory elastance will receive lower tidal volume compared to a patient with lower respiratory system elastance. Thus, during Co-MV, patients with differing elastance may not receive VT within the goal 6–8 mL/kg range, increasing the risk of volutrauma [20, 23, 24]. To ensure safe VT distribution, patients must be matched for elastance using a validated respiratory mechanics model able to identify this value and corresponding resistance.

In this research, we present a model-based method to help guide clinical decision-making in matching patients for in-parallel Co-MV, at least over short periods of time before patient state changes. This method extends existing Co-MV circuit recommendation through the identification of matching patients for Co-MV using patient-specific respiratory mechanics from a well-validated respiratory mechanics model. This method then uses simulation to determine the best set of MV settings to guide Co-MV care. Two outcomes are provided by the proposed model-based approach. First, the matching of suitable pairs of patients to undergo Co-MV. Second, determining the corresponding MV settings for Co-MV with one ventilator and two patients. In addition, a web application with a graphical user interface (GUI) has been developed and available online to accelerate the sharing of the proposed model.

## Results

### Pairing patient selection

**Fig. 1:**
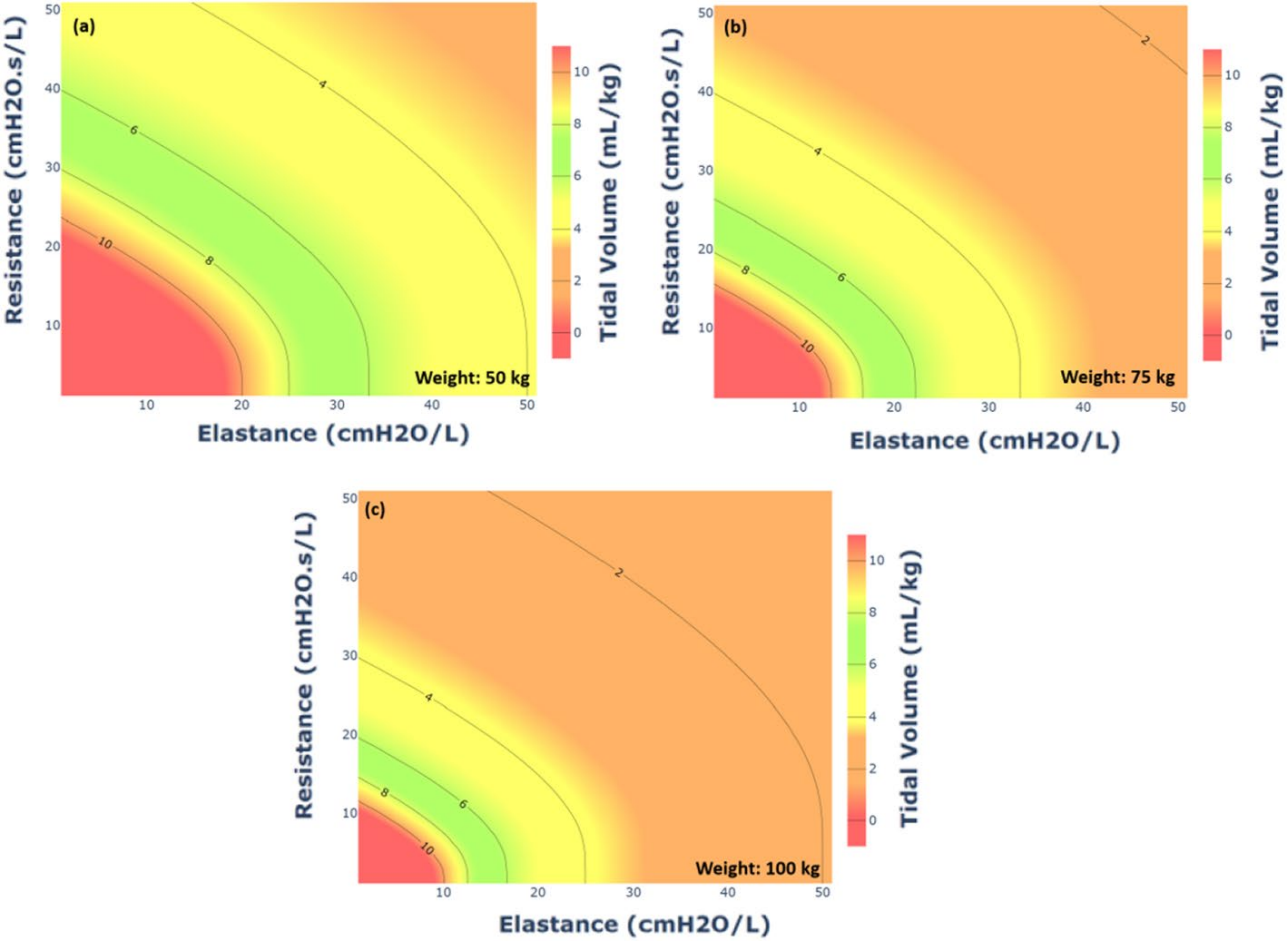
Resistance–elastance tidal volume contour plots for patient’s weight at 50 kg (**a**), 75 kg (**b**), and 100 kg (**c**)

The simulated tidal volume obtained from the single-compartment model (SCM) (refer to Methodology Eq. (1)) is presented in resistance–elastance tidal volume contour plots (R–E plot) in Fig. 1a–c, showing the distribution of VT based on different respiratory mechanics using the MV settings shown in Table 3 of the methodology section. Figure 1a shows the R–E contour plot for patient weighing at 50 kg, Fig. 1b at 75 kg and Fig. 1c at 100 kg.

The green zone is denoted as a safe zone for Co-MV where VT falls in the range of 6–8 mL/kg. The gradual change from green-to-yellow, yellow-to-orange, and orange-to-red zones represent changes from VT = 6–8 mL/kg to higher tidal volume (moving towards the bottom left of each figure), or to lower tidal volume (moving towards the upper right of each figure). Figure 1 also shows that the region of 6–8 mL/kg (centre green) reduces if a patient weight increases. For a 100-kg patient in Fig. 1c, the ranges of patient-specific E and R for Co-MV with Patient 1 are highly restricted, as expected due to a narrower range of safe tidal volume. At low resistance values in each figure, the curve is nearly vertical as the change in VT is primarily a function of elastance when R is low. As R increases, VT is more sensitive, as this term in Eq. (1) plays a greater role. Thus, the R–E contour plots are not only MV setting-specific, but are also patient weight-specific.

**Fig. 2:**
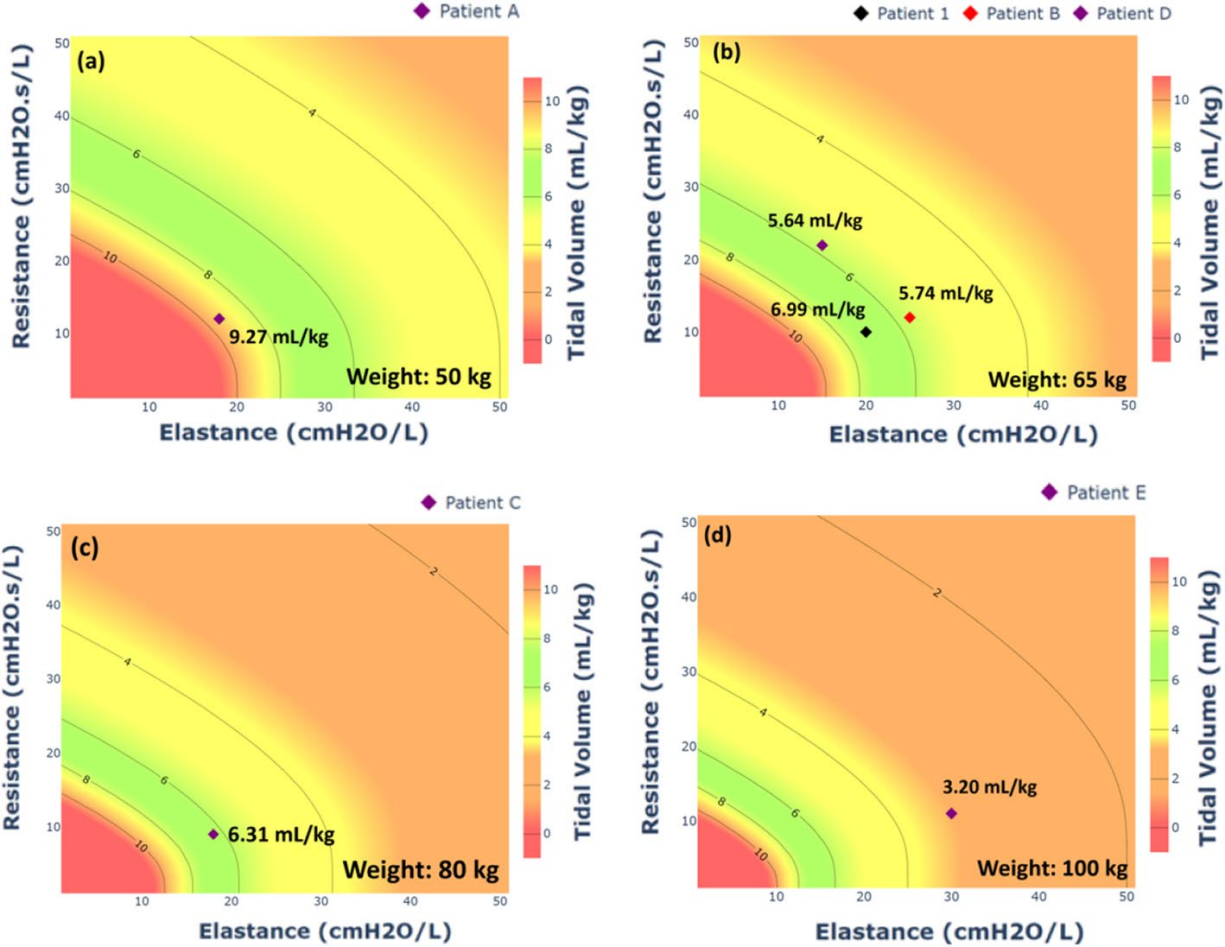
Resistance–elastance tidal volume contour plots for patient with 50 kg (**a**), 65 kg (**b**), 80 kg (**c**), and 100 kg (**d**). The estimated tidal volume for Patients 1, A, B, C, D and E are indicated in the respective weight-specific R–E plot

Figure 2a–d shows the contour plots for patient weights at 50 kg, 65 kg, 85 kg and 100 kg. The patients in Tables 3 and 4 are indicated in their respective weight-specific R–E plots. The VT for each patient in Tables 3 and 4 is presented using their corresponding weight. Based on Fig. 2b, the resultant VT of Patient 1 is ~ 6.99 mL/kg, and the VT of Patients B and D fall outside the green region with tidal volume lower than 6 mL/kg at 5.74 mL/kg (Patient B) and 5.64 mL/kg (Patient D). For Patient B with moderate respiratory failure, the lungs are stiffer (with higher E) than Patient 1, shifting them to the right. Equally, Patient D with significantly higher resistance is shifted upwards from Patient 1. For Patients B and D, higher inspiratory pressure is required to increase the volume of delivered air to the lungs.

Figure 2a shows the MV setting for mild respiratory failure patient is not suitable for Patient A with a weight of 50 kg as the VT exceeds 8 mL/kg. Equally, Fig. 2d shows Patient E who weighs 100 kg falls in the orange zone and should be eliminated from Co-MV candidates. The results of these two patients further highlight the importance of pairing patients with consideration of predicted body weights. Finally, in this study, the ideal patient to be paired with Patient 1 is Patient C, as shown in Fig. 2c with an estimated VT of ~ 6.31 mL/kg when delivered with the same MV settings in Table 3.

### Recommended MV settings

**Fig. 3:**
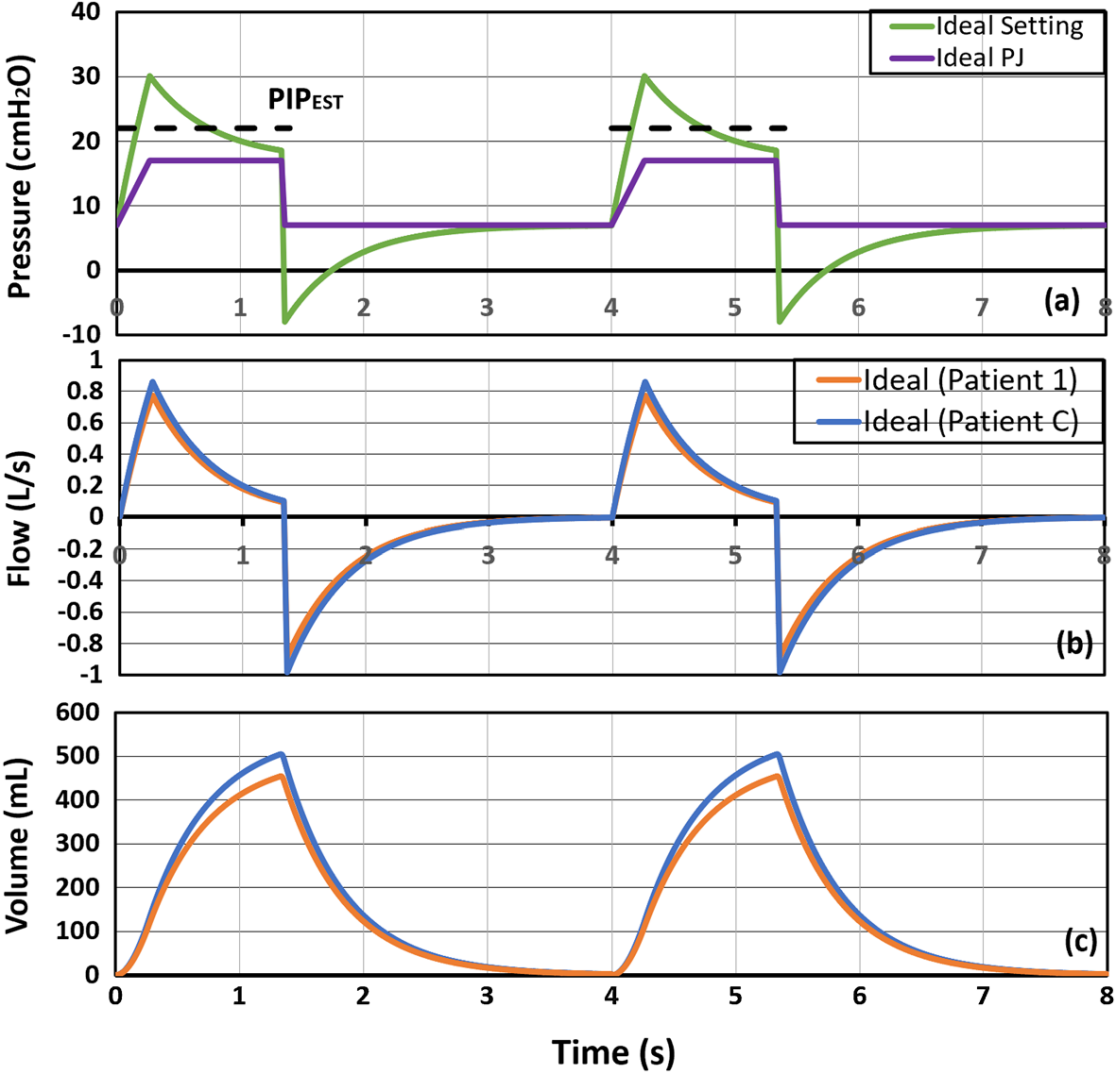
Ideal Co-MV settings and inputs for two breathing cycles. **a** Airway pressure during Co-MV; **b** flow for Patient 1 and Patient C, and **c** air volume for Patient 1 and Patient C

With a decision on pairing Patient 1 with Patient C, the DCM of Eq. (3) is simulated to obtain MV settings for Co-MV. Both Patient 1 and Patient C’s respiratory mechanics are used as input to the DCM model to simulate final Co-MV pressure control settings. For the simulation, an arbitrary value of 8 cmH2Os/L is assigned to RC of the Co-MV circuit to represent the potential increase of MV circuit resistance due to connection extension to two Patients. The ideal MV pressure settings for Co-MV with the initial inputs (PJ(t), V̇1(t), V̇2(t), V1(t), V2(t)) computed from SCM are shown in Fig. 3.

The ideal PJ(t) (purple line in Fig. 3a) represents the pressure graph generated from MV based on the initial MV setting in Table 3. A positive flow rate indicates air entering the lungs during inspiration, whereas a negative flow rate indicates the exiting airflow during expiration. Due to the existence of Rc, the resultant solution for P(t) has appeared to be an inverse ramp waveform (green line) as shown in Fig. 3a. Theoretically, this is the ideal airway pressure to be delivered by the MV for both Patients 1 and C for them to receive the desired VT. Unfortunately, modern pressure-controlled MV only generates square wave-like pressure. It is also implausible to apply a negative airway pressure. Thus, to ensure sufficient VT is delivered, the main factor to be considered is to adjust the inspiratory pressure generated from the ventilator, which can be achieved via simple MV settings.

**Fig. 4:**
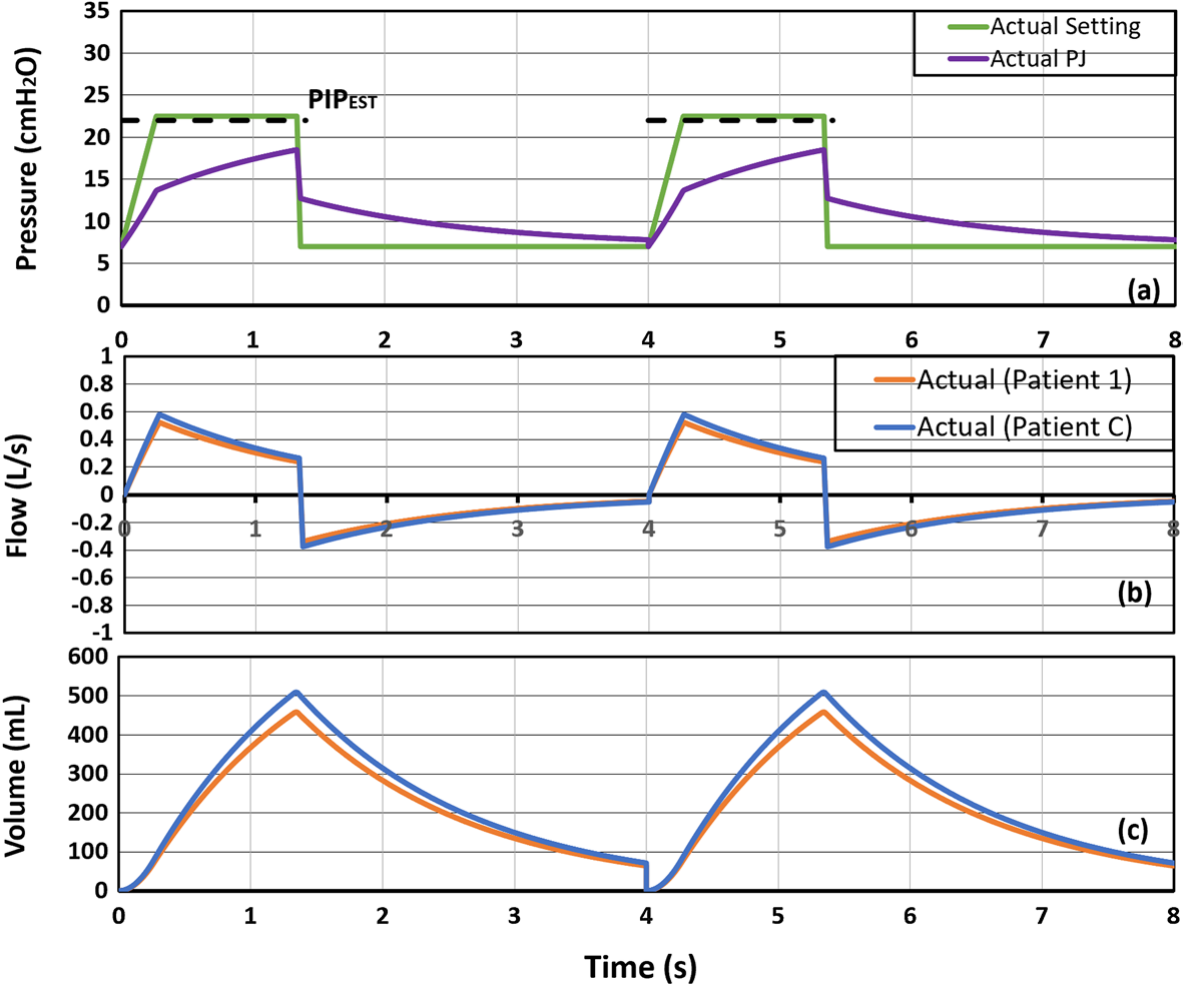
Proposed actual Co-MV settings for two breathing cycles with the estimated flow and volume: **a** airway pressure during Co-MV; **b** flow for Patient 1 and Patient C, and **c** air volume for Patient 1 and Patient C

In this study, the inspiratory pressure is controlled by changing the PIP while keeping the PEEP constant. Based on the ideal P(t), PIPEST is estimated by taking the average pressure from the ideal setting as shown in Fig. 3a (green solid line), resulting in the PIPEST as the dotted black line. However, this approach will typically lead to a lower VT compared to ideal values. In response, the proposed actual MV setting is obtained through an iterative process. The value of PIPEST obtained from the ideal setting is increased by 0.5 cmH2O for each simulation iteration before a VT with minimum error compared to the desired VT is obtained. Figure 4a (green solid line) shows the finalised actual MV pressure setting for Co-MV after several iterations. Based on the finalised MV setting, PIP has increased to 22.5 cmH2O, which is 5.5 cmH2O higher than the initial MV setting at 17 cmH2O. The inspiratory pressure required to deliver the desired VT to each patient is thus equal to 15.5 cmH2O (PIP–PEEP = 15.5 cmH2O). As a result, the actual PJ(t) is no longer a perfect square wave (purple solid line). Compared to the ideal PJ in Fig. 4a, the actual PJ has a higher peak inspiratory pressure.

The peak flow rate in Fig. 4b shows a significant decrease compared to the ideal flow of Fig. 3b. Nonetheless, the subsequent decreasing rate of the actual airflow is lower thus ensuring sufficient air is delivered to the lungs. From Fig. 4c, the ideal VT is 454.2 mL, whereas the actual VT is 458.5 mL, which is a negligible difference at 0.86% error. For the result of Patient C, the ideal and the actual VT are also very close at 0.95% error. The summarised results of VT in mL/kg are shown in Table 1.

**Table 1:** Results of VT for patients under Co-MV

	Patient 1	Patient C
Ideal VT (mL/kg)	6.99	6.31
Actual VT (mL/kg)	7.05	6.36
Percentage error (%)	0.86	0.95

## Discussion

### General implementation

The proposed protocol has demonstrated successful patient matching and MV setting in a simulated environment. Apart from finding a suitable patient, further noteworthy information can be obtained from this pairing process. First, due to the highly restrictive respiratory mechanics, obese patients have to be singly ventilated with extra care. This result was not noted in any prior simulation studies, nor in the one clinical case study done [8], and represents a major risk given rising obesity rates, and the greater risk during COVID-19 for patients with this comorbidity.

**Fig. 5:**
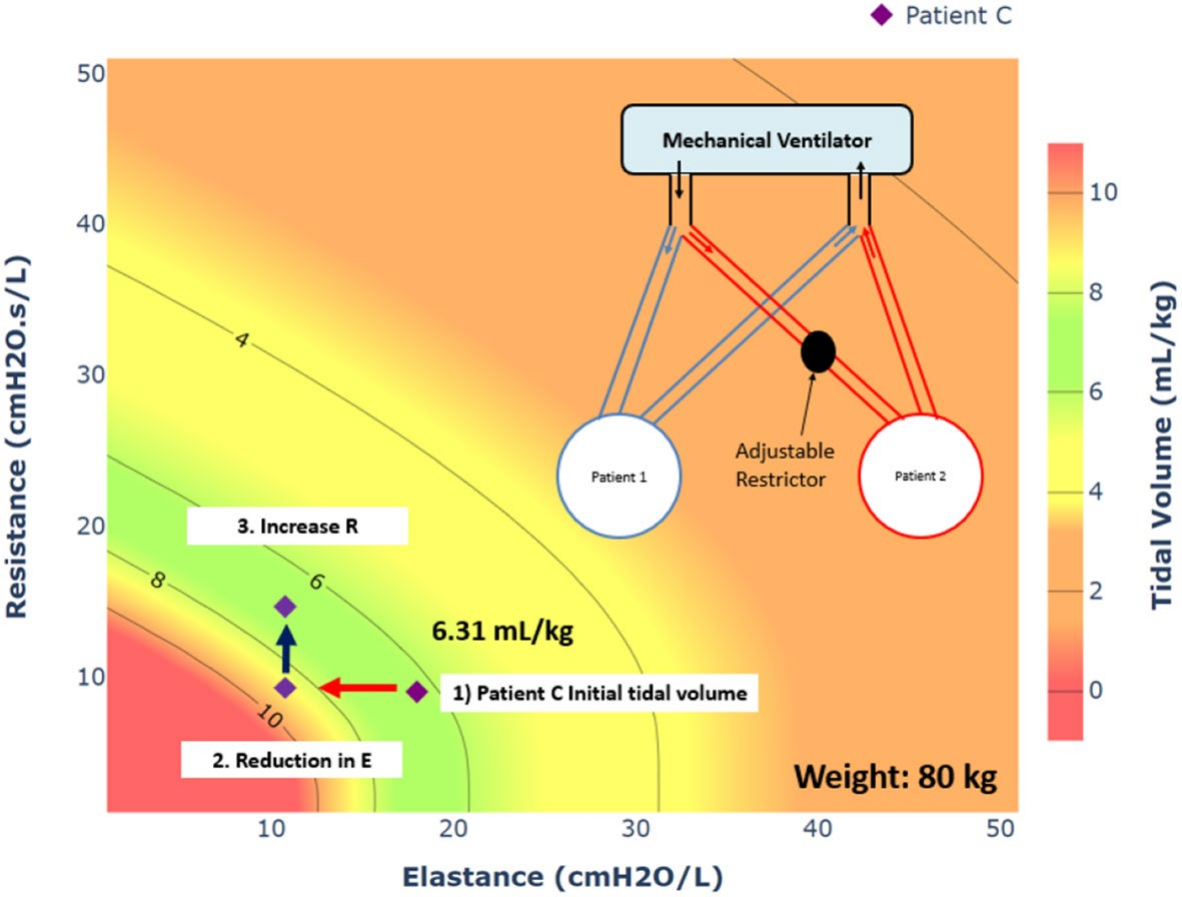
Resistance–elastance tidal volume contour plots for patient weighing 80 kg. The placement of the adjustable resistor in the parallel Co-MV circuit (Patient C) is shown in the upper right corner. As Patient C’s condition improves with lowering of respiratory elastance (E), the tidal volume delivered increases and moved out of the safe green zone. With the increase of resistance (R) in Patient C’s Co-MV circuit, the tidal volume range can be shifted back to the green zone

Aside from providing the estimated VT, the R–E contour plots can provide insights for the clinicians to evaluate the subsequent effects of Co-MV. The R–E tidal volume contour plot enables adjusting patients MV circuit resistance to increase or decrease tidal volume. For example, increasing the airway resistance can shift the VT of Patient A lower so it falls within the green zone. This resistance can be achieved by inserting an adjustable resistor in the inspiratory tube. The added resistor component will thus lower the amount of tidal volume entering Patient A. Such resistors can be placed in the respiratory circuit at the start and left at very low or zero value until needed. Another example is if Patient C has clinically improved during the process of Co-MV and the value of E drops to 10 cmH2O/L, their VT is now out of the green zone and might place the patient at risk. With this foreseeable situation, the clinician can be given an alternative to instal an adjustable resistor in the inspiratory path of Patient C as shown in Fig. 5. This resistor can decrease the potential tidal volume delivered to Patient C.

### Future work

Using the model-based approach, clinicians are able to virtually preview the results of Co-MV by pairing patients in a virtual environment. Similar to the DCM model of Eq. (3), which is also novel to this work, different settings can be tested virtually without causing harm. Nevertheless, this study is conducted within a simulated environment by implementing a purely mathematical model. Experiments must be performed in vitro, followed by animal and/or clinical studies to further validate the efficacy and safety of the proposed model-based method. However, there is no specific physiological reason the system could not work as shown. In this study, the proposed method is compared to a clinical Co-MV study performed by Beitler et al. [8]. The patient-specific information and the ventilator settings from the clinical study are used as input for the proposed method. The resulting tidal volume received by each patient during Co-MV from Beitler et al. is compared to our proposed model simulation. Results show that our simulation yields an average difference of 11.57% in the VT compared to Beitler et al. study (please refer to Additional file 1 for the details). We also found these Co-MV patient pairs, if ventilated with a specific ventilator setting, they are within a safe tidal volume range similar to the clinical trial. These results, based on comparison to the one clinical study of patient pairing to date, further highlight the potential of this proposed method in better matching Co-MV patients.

Setting MV for respiratory failure is difficult and it is even cumbersome during Co-MV. Thus, additional MV adjustment factors should be considered in real-life application. For instance, one patient might need a higher fraction of inspired oxygen (FiO2) to increase oxygenation when collapsed alveoli are not recruitable, which is not possible with either in-parallel or in-series Co-MV. The proposed model is unable to provide the adjustment required for the remaining MV parameters other than inspiratory pressure during pressure-controlled Co-MV. Equally, patient-specific parameters are likely to diverge over time as the disease progresses differently for each patient. Such inter- and intra-patient variability is a major concern in any form of MV [25, 26]. To overcome the inconsistencies of respiratory system mechanics, the proposed method can be implemented in a closed-loop system capable of monitoring the health conditions of the Co-MV patients in real-time [22, 27–29]. An in-series that enables more control and personalised MV, which would also benefit from patient matching, could be used where each patient has a unique inspiratory circuit and control [30].

Clinically, although the proposed method has demonstrated the ability to assist clinicians during Co-MV, Co-MV should only be treated as a temporary solution and last resort during a health crisis [12]. Thus, any use of this ventilation approach should be restricted or held for short term MV until more capacity can be found. Equally, COVID-19 and respiratory failure patients could be avoided for Co-MV, where more stable patients requiring MV could be matched, thus reducing some of the potential issues with variability.

### Open access

To accelerate the sharing of the proposed model amid the COVID-19 pandemic, a web application with a graphical user interface (GUI) has been developed by using an open-source programming language, Python. Flask is chosen as the web framework for web app development. In terms of the graphical presentation, the Plotly Python graphical library is implemented. The web application is developed based on the proposed virtual protocol and can be found in the following link (https://multiplex-ventilation.herokuapp.com/pressurecontrol). The web application summary and guide are included in an Additional File 1, along with this manuscript.

## Conclusions

Deciding the patients for co-ventilation is a complex and stressful process. The model-based approach presented in this study could serve as a guideline to determine a pair of patients and the corresponding MV settings for in-parallel co-mechanical ventilation. By having preliminary results prior to practical application, the risk of causing catastrophic complications and VILI can be decreased significantly. Nevertheless, additional clinical trials are required for further validations.

## Methods

### Setting up for co-ventilation (Co-MV)

In this study, a set of ‘safe’ PC MV settings is used based on several major clinical studies, as shown in Table 2.

**Table 2:** Recommended MV settings from literature

Parameters	Criteria	Clinical study references
Plateau pressure, PPLAT	< 35 cmH2O	Gattinoni et al. [31]
Positive end-expiratory pressure, PEEP	5–25 cmH2O	Gattinoni et al. [32]
Respiratory rate, RR	12–20 breath per min	O’Driscoll et al. [33]
Inspiratory expiratory ratio, I:E	1:2–1:5	Poor et al. [34]
Tidal volume, VT	6–8 mL/ kg	ARDSNet [35]

**Fig. 6:**
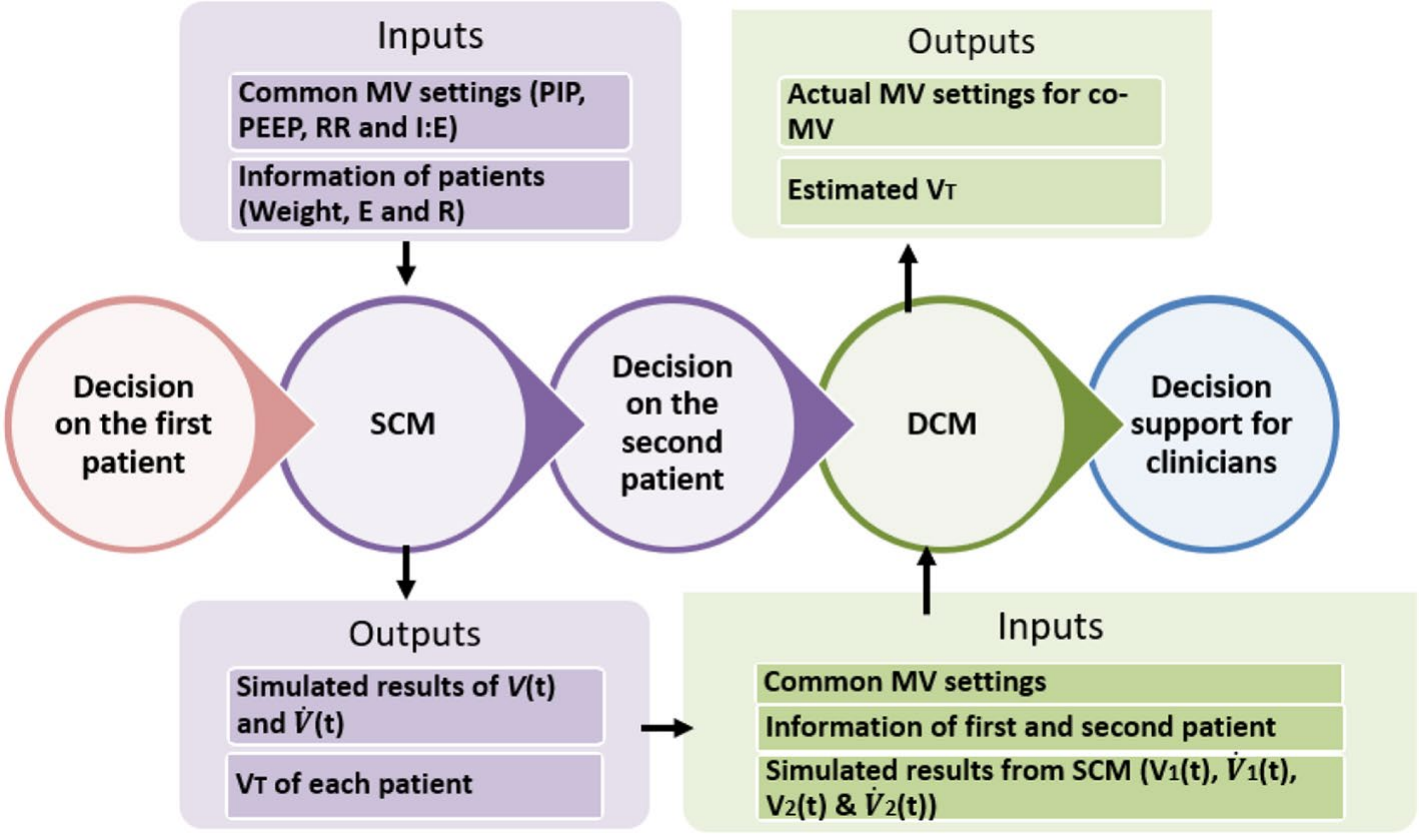
Flowchart of the protocol stages for Co-MV

To set up Co-MV, a patient must first be ventilated to obtain the corresponding patient-specific respiratory mechanics, airway resistance (R) and respiratory system elastance (E). These values can provide information on their response to MV and can be identified from measured airway pressure and flow using multiple linear regression [26, 36]. A clinically validated single-compartment linear lung model (SCM) [26, 36–38] is then used together with the respiratory mechanics to forward simulate potential mechanical ventilator output. The output can then assist clinicians in selecting the most compatible patients for Co-MV. After finding the suitable patient pair, further simulation using a double-compartment linear lung model (DCM) is conducted to determine the best Co-MV settings for these two specific patients. The summary of performing Co-MV is shown in the flowchart in Fig. 6.

### Lung compartment models

**Fig. 7:**
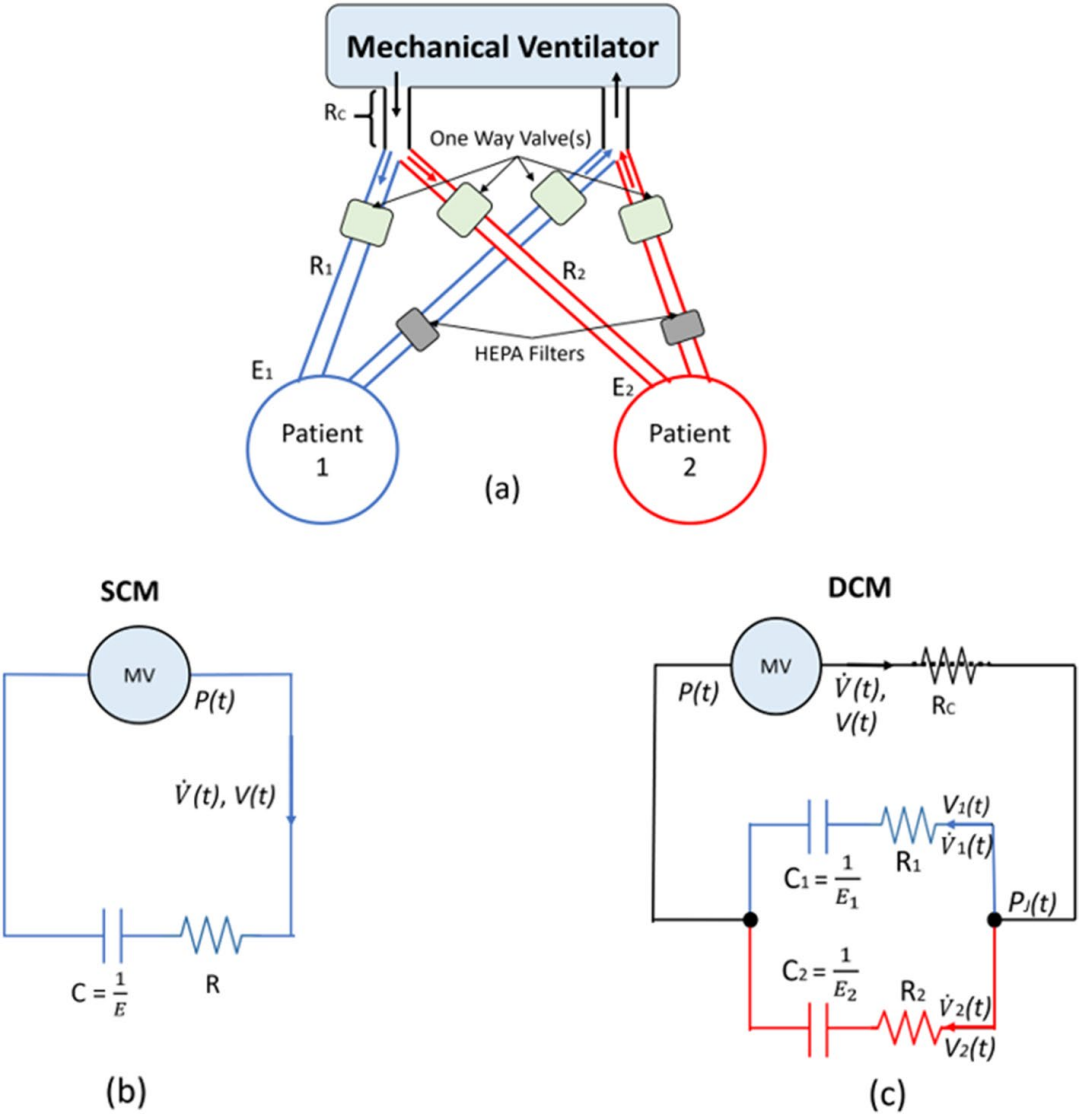
Setup for Co-MV (**a**) and the equivalent mathematical model for single-compartment model (SCM) (**b**) and double-compartment model (DCM) (**c**)

A sample setup for in-parallel Co-MV and the equivalent mathematical models to represent the Co-MV setup is shown in Fig. 7. The ventilator circuit (Fig. 7a) can be separated into two circuits in-parallel to connect to two patients. Each patient circuit, Patient 1 (blue) or Patient 2 (red) are fitted with one-way valves to ensure single direction flow. At each expiration circuit, they are fitted with HEPA filters to prevent cross-contamination. Figure 7b shows single-patient ventilation represented by a SCM and Fig. 7c shows the DCM represented an in-parallel Co-MV circuit.

The mathematical model of a single-patient MV circuit can be represented by an electrical circuit as shown in Fig. 7b describing the single-compartment lung model [39]:

1 $$P\left(t\right)=EV\left(t\right)+R\dot{V}\left(t\right)+{P}_{0},$$

2 $$\dot{V}\left(t\right)+\frac{EV\left(t\right)}{R}=\frac{(P\left(t\right)-{P}_{0})}{R},$$

where the airway pressure (P) is controlled during PC ventilation mode, time (t), volume (V), and flow ($\dot{V}$) are measured from the ventilator. The respiratory elastance (E), airway resistance (R) can be determined via multiple linear regression with P0 assumed as positive end-expiratory pressure (PEEP) when there is no intrinsic PEEP.

For the double-compartment model DCM version, as shown in Fig. 7c, where the second patient shares the input pressure. In the electrical circuit, compliance (C) is the inverse of elastance (E). The parallel circuit for Co-MV with a second patient is thus defined:

3 $$ \left( {R_{1} + R_{2} } \right)\dot{P}\left( t \right) + \left( {E_{1} + E_{2} } \right)P\left( t \right) = \left[ {R_{1} R_{2} + R_{c} \left( {R_{1} + R_{2} } \right)} \right]\ddot{V}\left( t \right) + \left[ {\left( {R_{c} + R_{2} } \right)E_{1} + \left( {R_{c} + R_{1} } \right)E_{2} } \right]\dot{V}\left( t \right) + E_{1} E_{2} V(t), $$

where Patient 1 and Patient 2 are defined in terms of their patient-specific respiratory mechanics (E1, E2, R1, R2) and resulting patient-specific volume and flow (V1(t), V̇1(t), V2(t), V̇2(t)) using subscripts, and are seen in the blue and red paths in Fig. 7c. RC is the common resistance component due to the ventilation circuit where it is shared during Co-MV. PJ is the pressure at the joint connecting both patients during Co-MV. Equation (3) can be solved to obtain the resulting behaviour, where all simulations are performed using MATLAB 2019 (Natick, MA).

### Simulation of Co-MV patient pairing

In this study, we demonstrate the model-based pairing process through simulation of virtual patients [40–43]. A virtual patient, Patient 1 is simulated as the first candidate considered for Co-MV. Table 3 shows Patient 1’s respiratory mechanics and the corresponding PC mode MV settings used for their MV care. Five additional virtual patients, Patients A, B, C, D and E with different respiratory mechanics are assigned as potential candidates for Co-MV. These patients’ suitability to be paired with Patient 1 are evaluated using model-based predicted tidal volume (in mL/kg) corresponding to patient-specific respiratory mechanics and weights. Solving Eq. (2) with P(t), P0, R and E, and patient weight as input will enable estimation of each patient’s predicted tidal volume.

**Table 3:** Patient 1 respiratory mechanics and MV settings

Patient ID	Respiratory failure	E (cmH2O/L)	R (cmH2Os/L)	Weight (kg)
1	Mild	20 (18–27)	10 (9–14)	65

Initial MV settings for Patient 1	
PIP (cmH2O)	17
PEEP (cmH2O)	7
RR (bpm)	15
I:E	1:2

To ensure the patient-specific values of E and R are realistic, they are chosen based on the values found in the literature [44]. Table 4 shows the respiratory mechanics of the 5 potential virtual patients (A, B, C, D, E) screened for Co-MV with Patient 1. Each patient’s respiratory mechanics were set based on different levels of respiratory failure, where a severe respiratory failure results in greater elastance, and obstructive disorders have greater resistance. In this study, the mean values of the patient’s respiratory system elastance and resistance are used.

**Table 4:** Potential patients to be Co-MV with Patient 1

Patient ID	Respiratory failure	E (cmH2O/L)	R (cmH2Os/L)	Weight (kg)
A	Normal	18 (15–22)	12 (10–15)	50
B	Moderate	25 (20–32)	12 (10–14)	65
C	Mild	18 (18–27)	9 (9–14)	80
D	Obstructive	15 (13–23)	22 (16–33)	65
E	Severe	30 (22–33)	11 (9–14)	100

Patient 1 with mild respiratory failure, is the first patient being ‘ventilated’, with Patients A–E being potential matches. With the common MV setting from Patient 1, and the information of each patient, simulation is conducted with Eq. (2) to select the second patient from among Patients A to E. For a more general solution, values of E and R within the range of 1 to 50 will be simulated for the second patient to assess where the best patient match might be. After selecting the second patient, the final step is to simulate the actual MV setting by using the DCM model of Eq. (3). The feasibility of the actual MV setting is evaluated by comparing the percentage difference of VT when co-ventilated.

## Supplementary Information

**Additional file 1. **Web application.

## Data Availability

Data are available upon request.

## Abbreviations

C: Compliance
Co-MV: Co-mechanical ventilation
DCM: Double compartment lung model
E: Respiratory elastance
FiO2: Fraction of inspired oxygen
GUI: Graphical user interface
HEPA: High efficiency particulate air
I:E: Ratio of inspiration time to expiration time
MV: Mechanical ventilation
P: Airway pressure (P)
P0: Offset pressure
PC: Pressure control
PEEP: Positive end-expiratory pressure
PIP: Peak inspiratory pressure
PIPEST: Estimated peak inspiratory pressure
PJ: Pressure at the joint connecting both patients
PPLAT: Plateau pressure
R: Airway resistance (R)
R–E: Resistance–elastance
RR: Respiratory rate
SCM: Single compartment linear lung model
t: Time
V: Volume
$$\dot{V}$$: Flow
$$\ddot{V}$$: Acceleration
VILI: Ventilator-induced lung injury
VT: Tidal volume
