# Supplementary material for: Model-based patient matching for in-parallel pressure-controlled ventilation
Source: Biomed Eng Online. 2022 Feb 9;21:11. doi: 10.1186/s12938-022-00983-y (PMC8826717; doi:10.1186/s12938-022-00983-y)
Supplement: Supplementary file 1 — Additional file 1. Web application. [file 12938_2022_983_MOESM1_ESM.docx]

**Additional File A – Web Application**

**1.0 Web application interface and guide**

This section will introduce the interface of the web application in detail, along with the additional functions implemented. At the start of the application, the users are required to select the first patient to undergo Co-MV, which is represented by “Current Patient” in the software. For the example used in the manuscript, Ventilator settings are 1) Respiratory rate =15 breaths per minute, 2) PEEP = 7 cmH_2_O, 3) PIP = 17 cmH_2_O and 4) I:E ratio of 1:2 = 0.5. Current patient is set as Patient 1, Weight = 65 kg, Elastance = 20 cmH_2_O/l and Resistance = 10 cmH_2_Os/l as shown in Figure A1.


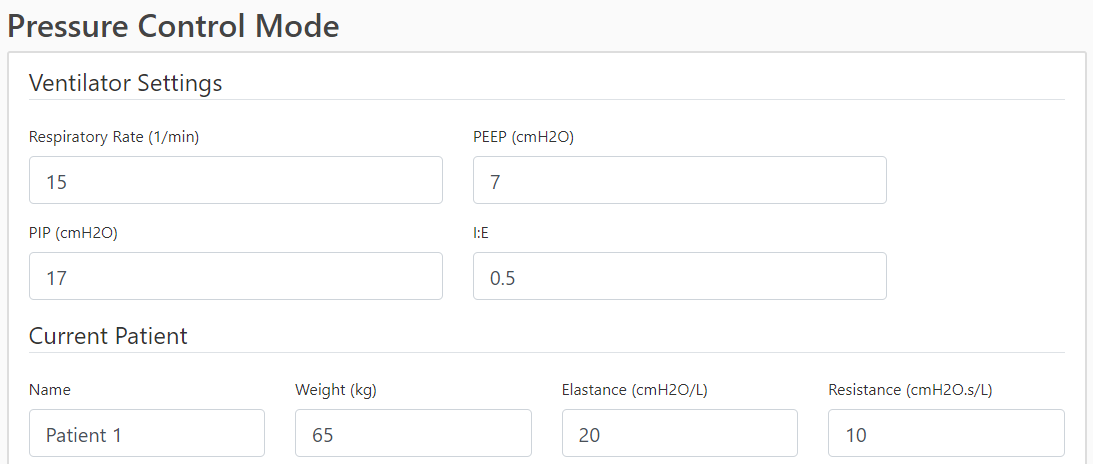


Figure A1. Entering ventilator settings and first patient for Co-MV in the user interface.

Next, the software will prompt the users to enter the information for potential pairing patients. The information of the candidates will be added in the form of a table, and it can be further customised by the clinicians by using the “Add” and “Delete” function. Examples used in the manuscript are added and is shown in Figure A2.


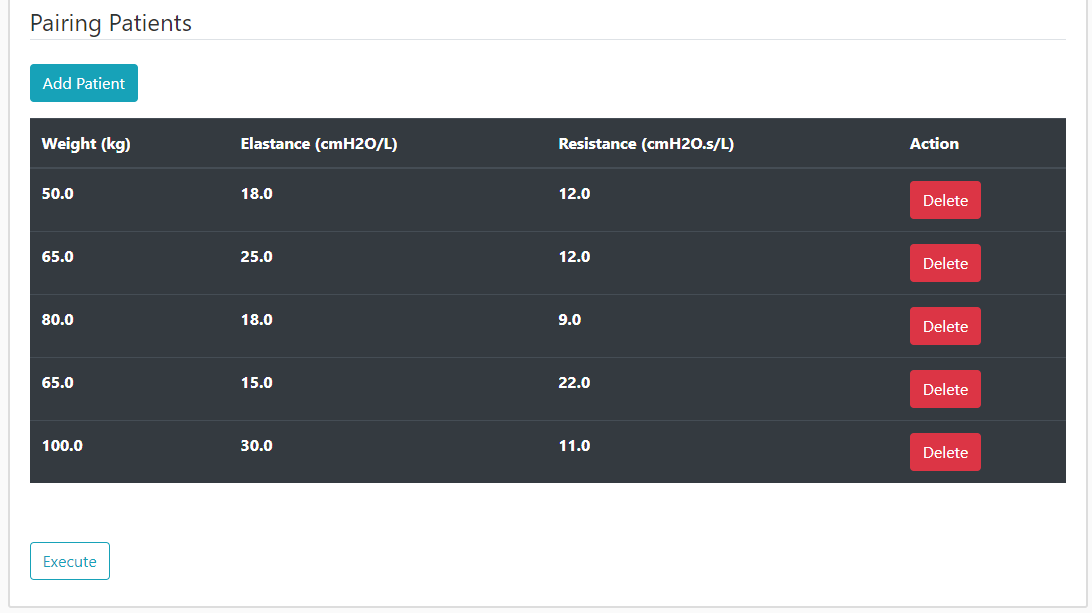


Figure A2. Adding potential pairing patients into user interface.

Once the potential patient for Co-MV are included, click the ‘Execute’ button. Upon simulation, graphs and table showing the estimated ventilator output will be displayed as shown in Figure A3. For validation purpose, the simulated ventilator graphs for the current patient can be used as a comparison with the actual ventilation output.


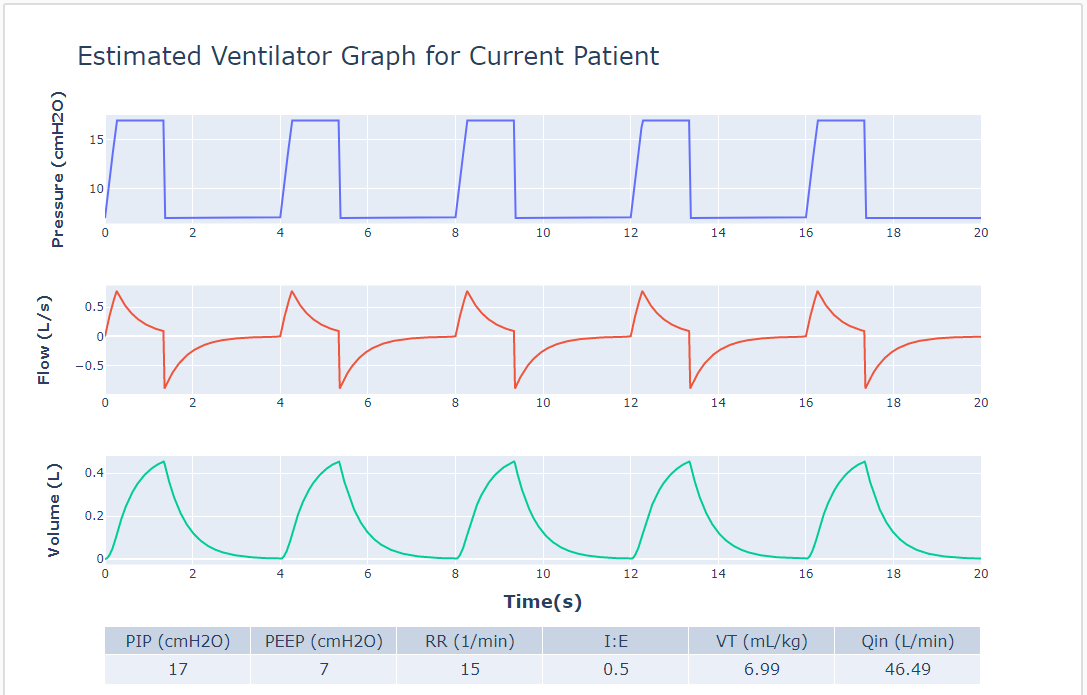


Figure A3. Estimated ventilator output of the current patient (Patient 1).

Figure A4 shows the contour plots of each potential pateint for pairing purposes. Note that the slider at the bottom of Figure A4 is used to switch between different patient whereas the table above shows the piecewise data in the green zone. In this case, Patient C, along with the corresponding estimated tidal volume (black rhombus) is shown in the R-E contour plot.


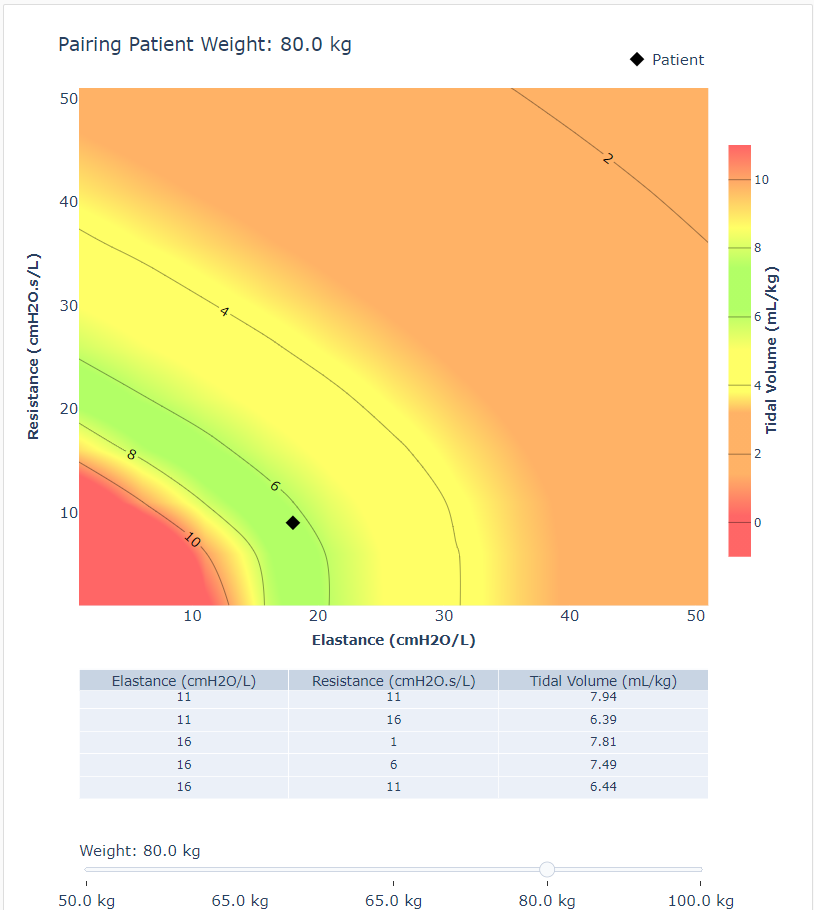


Figure A4. R-E contour plot for Patient C, 80 kg, E = 18 cmH2O/l, R = 9 cmH_2_Os/l.

As the final step, the users are required to fill up the form in Figure A5 to confirm which patient to be paired. An estimated common resistance of the Co-MV circuit must be entered before confirmation.


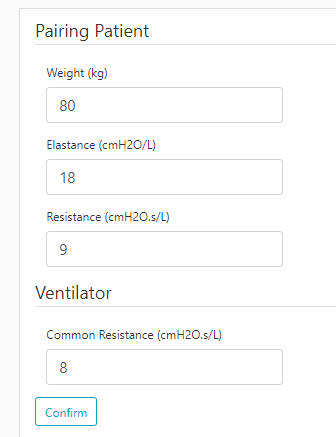


Figure A5. Confirmation on the second patient.

At the final stage, the software will display the simulation results regarding the recommended Co-MV setting and the estimated outcome during the Co-MV process of the selected patients. Figure A6 shows the summary of the recommended MV setting and the estimated tidal volume for each patient. The estimated tidal volume should fall within the range of 6-8 mL/kg.


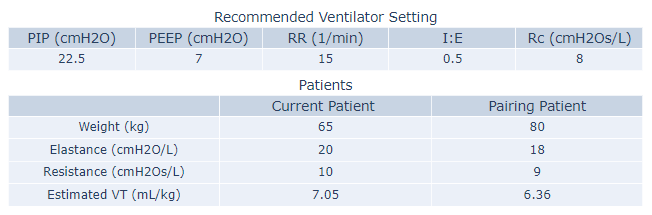


Figure A6. Summary of the recommended Co-MV setting and estimated tidal volume for each patient.

Following the tables, two graphs will be presented as shown in Figure A7. Figure A7 (Left) shows the estimated total pressure, flow, and volume of the MV. As for Figure A7(Right), the graphs present the estimated pressure, flow and volume of the air delivered to each patient during Co-MV. Additional sensors should be added in the real-world application in order to obtain the actual results for comparison and validation.


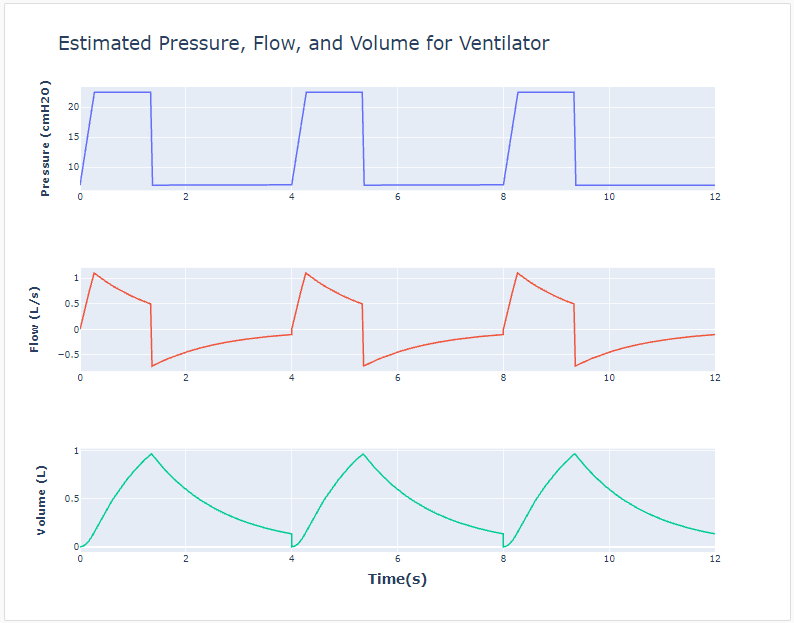

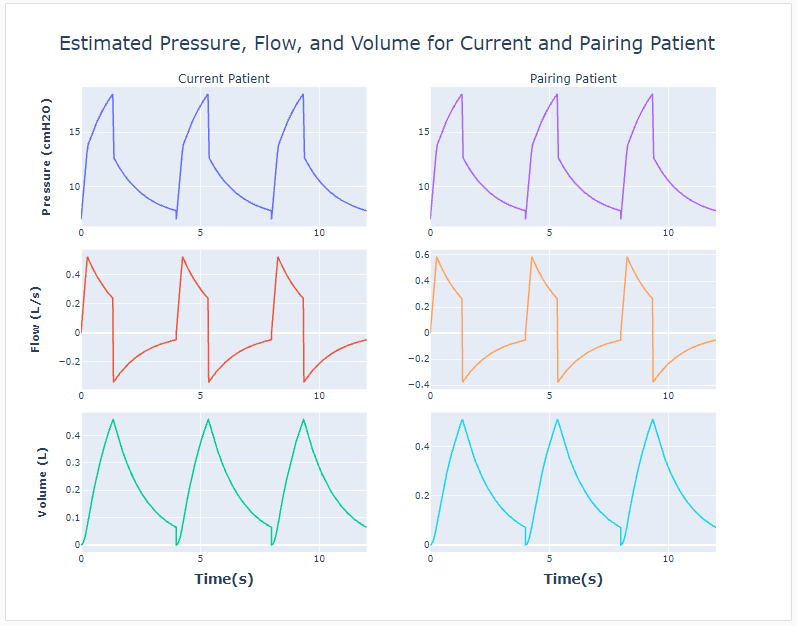


Figure A7. (Left) Estimated total pressure, flow, and volume of the Co-MV. (Right) Estimated pressure, flow and volume of the air delivered to each patient.

**2.0 Application process and validation**

Figure A8 shows the flow chart of the using the web application. The structure of the web application is categorised into four different stages. The task at each stage as shown in Table A1 is necessary to be completed before proceeding to the next stage to ensure the accuracy of the simulation results.


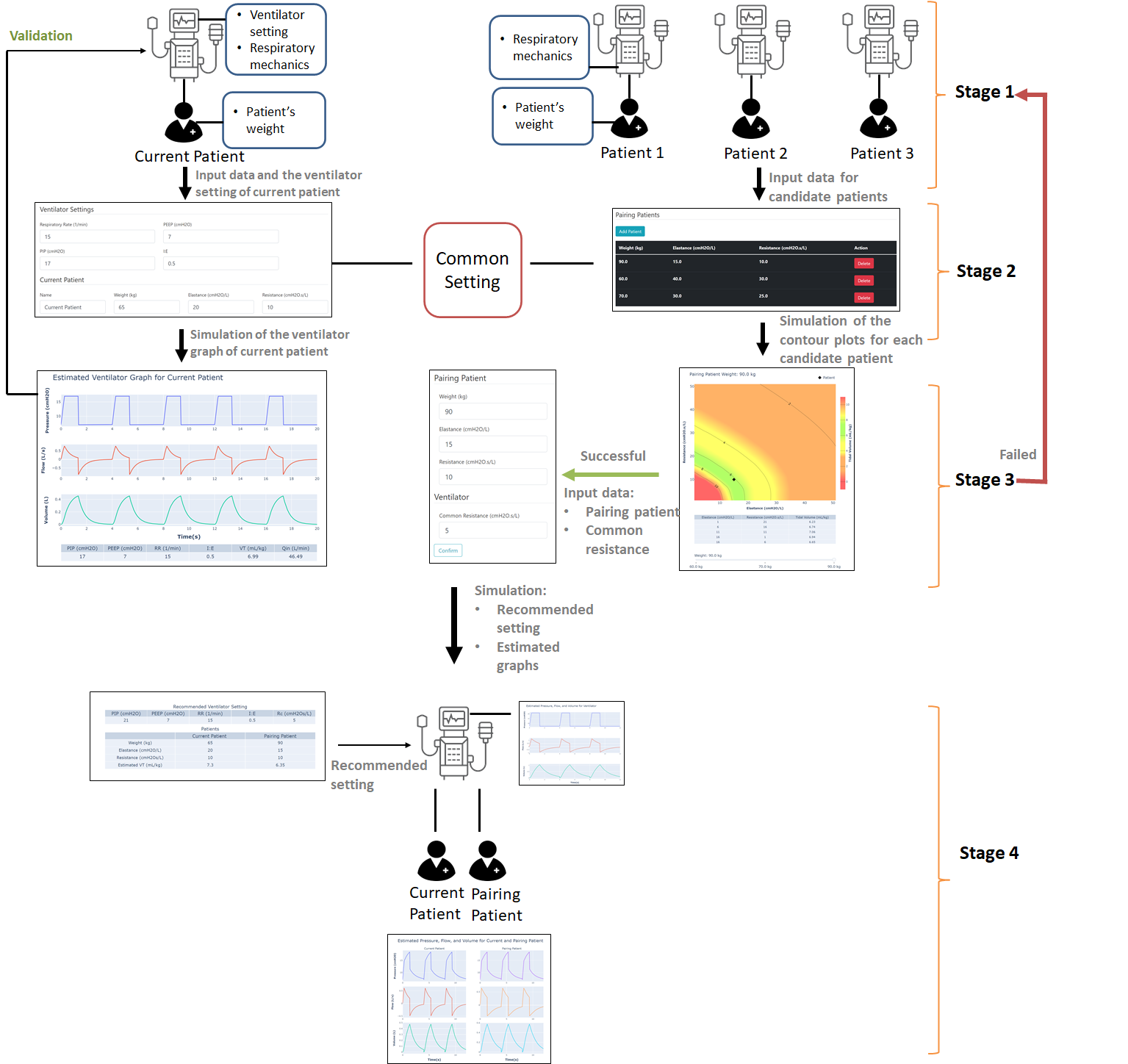


Figure A8. Software architecture of the web application.

**Table A1. Process for each stage**

| Stage 1 | Data Collection |
| --- | --- |
| Stage 2 | Data Input |
| Stage 3 | Pairing Patient |
| Stage 4 | Co-MV Setting |

Prior to initialising the app, data collection must be completed at Stage 1. As the initial step, the users are required to select the first patient to undergo Co-MV, which is represented by “Current Patient” in the software. Upon that, clinicians are required to select the potential patients to pair with the current patient, as represented by Patient A, B, C, D and E in the case examples shown in manuscript. Our goal is to select one of the candidates to pair with the current patient, while maintaining the corresponding ventilator setting. Therefore, aside from the respiratory mechanics and the weights of each patient, the recent MV setting for the current patient is required as well. Note that the mentioned MV setting is represented by the Common Setting (red box) in Stage 2. Upon data collection, the data has to be input to the web application at Stage 2. It is extremely crucial to ensure the correctness of the data prior to simulation. Simulation will be initiated after all the information required has been entered to the web application.

At Stage 3, a few graphs will be presented. These graphs can be used as validations and references for the clinicians to decide the second patient for Co-MV. By carrying out analysis based on the graphs, lung protective strategies can be planned to ensure the safety of both patients throughout the entire Co-MV process. In the case where none of the candidates are suitable for Co-MV, Stage 1 and Stage 2 have to be repeated with different candidate patients. Alternately, the Co-MV can be modified by implementing additional components, such as adjustable resistor and sensors.

After deciding the second patient, as the starting point of Stage 4, the app will recommend the adjustment requires for the MV setting. Mainly, the PIP setting of the ventilator need to be increased to overcome the built-up pressure due to the additional paths. On top of that, estimated outcome during Co-MV will be simulated, and presented in graphical form.

**3.0 Comparison with literature**

Patient data and MV setting information from Beitler et al. are used in a comparison analysis using the proposed method. Some model input assumptions are made using literature values if there is any insufficient data. For example, the common resistance (*R_C_*) during Co-MV for Beitler is not available and in this case, we assumed it to be 5 cmH_2_Os/L. The simulation results are compared to the results at 0 hour of the Co-MV by Beitler et al. The Co-MV settings, model input and simulation results are shown in Table A. It is noteworthy that Beitler et al. proposed a V_T_ range of 4-8 mL/kg, thus, we also adjusted the model recommended range to 4-8 mL/Kg for equal comparison.

Based on the results from our simulation, the overall percentage difference is around 11.57 %. Patient 2B had the highest difference compared to the literature results. On the other hand, V_T_ of Patient 3A has appeared to be the closest to the literature results. By implementing the proposed model, all 6 patients have successfully received a V_T_ within the recommended range. There is limited information regarding the setup of the co-ventilation circuit, and the real time situation during the co-ventilation process, which will highly affect the accuracy of the results. Hence, an average percentage difference of 11.57 % can be further reduced with more detailed information on hand. Aside from that, the proposed model has proven the suitability of these three pair of patients to undergo co-ventilation, which is consistent to the outcome of the literature study.

**Table A.** Results comparison

| **Patients** | **1A** | **1B** | **2A** | **2B** | **3A** | **3B** |
| --- | --- | --- | --- | --- | --- | --- |
| **Shared Ventilator Settings^1^** |  | |  | |  | |
| PIP (cmH_2_O) | 28 | | 37 | | 29 | |
| PEEP (cmH_2_O) | 12 | | 9 | | 9 | |
| Respiratory Rate (1/min) | 29 | | 29 | | 29 | |
| I:E Ratio | 0.5 | | 0.5 | | 0.5 | |
|  |  |  |  |  |  |  |
| **Model input^2^** |  |  |  |  |  |  |
| Predicted Body Weight (kg) | 54.80 | 77.80 | 50.10 | 66.10 | 61.60 | 84.70 |
| Respiratory Elastance, *E_rs_* (cmH_2_O/L) | 41.67 | 33.33 | 58.82 | 45.45 | 50.00 | 52.63 |
| Respiratory Resistance, *R_rs_* (cmH_2_Os/L) | 6.99 | 2.19 | 8.33 | 3.27 | 12.31 | 2.00 |
|  |  |  |  |  |  |  |
| **Results** |  |  |  |  |  |  |
| Actual Tidal volume *V_TA_*^3^ (mL/kg) | 5.90 | 4.80 | 8.20 | 5.80 | 5.00 | 4.80 |
| Simulated Tidal Volume *V_TS_*^4^ (mL/kg) | 5.34 | 5.15 | 7.78 | 8.02 | 4.96 | 4.06 |
| Difference between *V­_TA_* and *V_TS_* (%) | 10.49 | 6.80 | 5.40 | 27.68 | 0.81 | 18.23 |

^1^ Shared MV settings were extrapolated from Beitler et al. (Beitler et al., 2020). Assumptions were made if there is insufficient data.

^2^ Model input is based on calculation from respiratory compliance and single compartment model. Assumptions were made if there is no data.

^3^ Value is taken at the Zero hour, at the start of Co-MV in Beitler et al.

^4^ Tidal volume were obtained at common resistance at 5 cmH_2_Os/L.
